# Implementation of multigene panel testing for breast and ovarian cancer in South Africa: A step towards excellence in oncology for the public sector

**DOI:** 10.3389/fonc.2022.938561

**Published:** 2022-12-07

**Authors:** Nerina C. van der Merwe, Kholiwe S. Ntaita, Hanri Stofberg, Herkulaas MvE. Combrink, Jaco Oosthuizen, Maritha J. Kotze

**Affiliations:** ^1^ Division of Human Genetics, National Health Laboratory Service, Universitas Hospital, Bloemfontein, South Africa; ^2^ Division of Human Genetics, Faculty of Health Sciences, University of the Free State, Bloemfontein, South Africa; ^3^ Office of the Dean, Economic and Management Sciences, University of the Free State, Bloemfontein, South Africa; ^4^ Interdisciplinary Centre for Digital Futures, University of the Free State, Bloemfontein, South Africa; ^5^ Division of Chemical Pathology, Department of Pathology, National Health Laboratory Service, Tygerberg Hospital, Cape Town, South Africa; ^6^ Division of Chemical Pathology, Department of Pathology, Faculty of Medicine and Health Sciences, Stellenbosch University, Cape Town, South Africa

**Keywords:** multigene panel testing, South Africa, oncology, next-generation sequencing, hereditary breast and ovarian cancer (HBOC) syndrome

## Abstract

Translation of genomic knowledge into public health benefits requires the implementation of evidence-based recommendations in clinical practice. In this study, we moved beyond *BRCA1/2* susceptibility testing in breast and ovarian cancer patients to explore the application of pharmacogenetics across multiple genes participating in homologous recombination DNA damage repair. This involved the utilisation of next-generation sequencing (NGS) at the intersection of research and service delivery for development of a comprehensive genetic testing platform in South Africa. Lack of international consensus regarding risk categorization of established cancer susceptibility genes and the level of evidence required for prediction of drug response supported the development of a central database to facilitate clinical interpretation. Here we demonstrate the value of this approach using NGS to 1) determine the variant spectrum applicable to targeted therapy and implementation of prevention strategies using the 15-gene Oncomine™ BRCA Expanded Panel, and 2) searched for novel and known pathogenic variants in uninformative cases using whole exome sequencing (WES). Targeted NGS performed as a routine clinical service in 414 South African breast and/or ovarian cancer patients resulted in the detection of 48 actionable variants among 319 (15%) cases. *BRCA1/2*-associated cancers were identified in 70.8% of patients (34/48, including two double-heterozygotes), with the majority (35.3%, 12/34) representing known South African founder variants. Detection of actionable variants in established non-*BRCA1/2* risk genes contributed 29% to the total percentage (14/48), distributed amongst *ATM*, *CHEK2*, *BARD1*, *BRIP1*, *PALB2* and *TP53*. Experimental WES using a virtually constructed multi-cancer NGS panel in 16 genetically unresolved cases (and four controls) revealed novel protein truncating variants in the basal cell carcinoma gene *PTCH1* (c.4187delG) and the signal transmission and transduction gene *KIT* (c.930delA) involved in crucial cellular processes. Based on these findings, the most cost-effective approach would be to perform *BRCA1/2* founder variant testing at referral, followed by targeted multigene panel testing if clinically indicated and addition of WES in unresolved cases. This inventive step provides a constant flow of new knowledge into the diagnostic platform *via* a uniquely South African pathology-supported genetic approach implemented for the first time in this context to integrate research with service delivery.

## Introduction

Breast cancer is the most common cancer affecting women worldwide. An estimated two million new cases will be diagnosed per year. (1, 2). The incidence is also increasing in South Africa, with an average rate of 1 in 32 for breast cancer development in females (3). Although most cases occur sporadically, 15–20% are associated with an inherited predisposition characterized by early-onset cancer in affected families (4). Translating population risk stratification to personal utility relies on evidence-based recommendations informed by the gene variant spectrum that may differ between countries. This is of particular relevance in South Africa due to a complex history of population migration over a period of 300 years, which resulted in varying degrees of founder pathogenic variants as detected in the two major cancer susceptibility genes, *BRCA1* and *BRCA2* (5, 6). These migration events included European colonialism, expansion of Africans to the south, and the introduction of laborers from southern Asia (https://southafrica-info.com/people/south-africa-population/). Collectively, when entwined with indigenous populations already residing at the most southern tip of the African continent, a unique genetic architecture evolved through mutational events and founder effects characteristic of the South African population comprising approximately 61 million people, being neither culturally, linguistically nor genetically homogenous.

Detection of pathogenic variants with varying penetrance in the *BRCA1* and *BRCA2* genes are responsible for approximately two-thirds of hereditary breast and ovarian cancer, with cumulative risks ranging from 16–84% and 11–87% for *BRCA2* and *BRCA1*, respectively, by age 80 years (7). The most extensive *BRCA1/2* series reported to date in the public sector of South Africa (*n*=1 429) revealed a positivity rate of 9.2% (*n*=137) actionable variants detected, with the majority (74%, 241 of 326) identified as founder variants (8). Despite the increase in throughput using next-generation sequencing (NGS) for *BRCA1/2* variant detection, less than 10% of patients with a personal history of breast and/or ovarian cancer received a positive test result. International studies furthermore indicated that for many genes, the evidence of an association with breast cancer is weak or imprecise, while reliable subtype-specific risk estimates are lacking. This led to large population studies such as those performed by the Breast Cancer Association Consortium (BCAC), which collectively screened more than 60 000 cancer patients and 53 000 control individuals for pathogenic variants in 34 breast cancer susceptibility genes (9).

Based on the estimated overall risk for breast cancer protein-truncating variants, *ATM*, *BRCA1*, *BRCA2*, *CHEK2* and *PALB2* were identified as the major cancer susceptibility genes in the study population (9). The risk implications of *TP53* characterized by predominance of causative missense variants, as well as *BARD1*, *RAD51C, RAD51D*, *PTEN, NF1* and *MSH6*, were defined as modest. Among these 12 genes, *ATM* and *CHEK2* had a stronger association with estrogen receptor (ER)–positive than with ER-negative breast cancer. In the case of *BARD1, BRCA1*, *BRCA2, PALB2, RAD51C*, and *RAD51D*, the odds ratios were higher for ER-negative breast cancer than for ER-positive breast cancer. Among the genes that had no evidence of a weak association with breast cancer overall, *FANCM* showed association with ER-negative breast cancer and *FANCC* with triple-negative breast cancer. Notably, the risk categorization of *ATM* changed from a moderate- to a high-risk gene, whereas the gene encoding for the cell-cell adhesion protein E-cadherin (*CDH1*) lost its place as a high-risk gene when based on the relationship between gene variant and protein expression data presented by the BCAC. Representation of the histopathology and tumour subtypes routinely assessed as therapeutic biomarkers may therefore affect risk classification, as also evidenced by the clinical relevance of pathogenic germline *CDH1* variants to invasive lobular breast cancer, but not ductal carcinoma of no special type. These findings underscore the potential value of pathology-supported genetic testing as a relatively new concept applied in breast cancer risk stratification with genetic counselling support (8, 10, 11), to help bridge the clinical interpretation gaps identified in Africa (https://www.aasciences.africa/publications/policy-paper-framework-implementation-genomic-medicine-public-health-africa). Most of the work in cancer genomics are performed in the context of research, but to move from basic research to translation requires feasibility and health economic studies performed in parallel to assay validation.

Evidence provided by consortia such as BCAC resulted in multigene panel screening, which together with whole exome/genome sequencing (WES/WGS), is becoming standard practice internationally. Genome-scale sequencing analysis and data sharing between African counties enable translational research involving return of results to eligible patients (12). We first reported the value of experimental WES in relation to a previously detected *BRCA1* variant of uncertain clinical significance (VUS) in a patient with a novel pathogenic *PALB2* variant, subsequently confirmed by Sanger sequencing (13). In another South African patient with recurrent breast cancer, WGS supported the discovery of a unique *TP53* germline variant as the most likely cause of breast and other cancers in the family (14). These findings justified the establishment of a cancer genomic database and patient registry to optimise the clinical management of individual patients at the intersection of research and service delivery (https://console.virtualpaper.com/stellenbosch-university/research19/#46/). Due to high costs and current limited interdisciplinary access to this resource, multigene panel testing was earmarked as a cost-effective diagnostic solution to reduce the cancer burden in South Africa. Lack of international consensus regarding the optimal number of genes to screen based on their clinical relevance and evidence-level for prediction of treatment response in breast and ovarian cancer patients, necessitated the development of a multi-assay platform for application of personalized genomic medicine (15). The high cumulative genetic risks incurred by pathogenic *BRCA1/2* variants paved the way for rapid founder variant testing as the first step to increase access to cancer genetic testing in South Africa and to reduce loss to follow-up (8, 16). The next step towards excellence in surgery and oncology involves multigene panel testing to determine the variant spectrum in additional moderate- to high-risk genes underlying breast and ovarian cancer in South African patients.

Targeted multigene panel testing involves the screening of various established hereditary breast and ovarian cancer genes as described above, simultaneously at costs often reported as lower than testing for *BRCA1* and *BRCA2* alone (17). Implementing such a panel including high- to moderate-risk genes involved in homologous recombination DNA damage repair (18) could hold great promise to maximize health benefits in a developing country. The potentially benefit lies not only in explaining *BRCA-*negative inherited breast and ovarian cancer, but also the identification of cancer patients and at-risk family members eligible for clinical interventions, surveillance screening, targeted therapy and potential prevention strategies (19). We, therefore, evaluated the relevance of the commercially available Oncomine™ BRCA Expanded Panel including 15 genes targeting the homologous recombination DNA damage repair pathway in parallel to WES as a discovery tool. If supported by our data, the integration of research and service delivery will improve the efficiency of breast and ovarian screening in South Africa, while at the same time increase our knowledge of genes involved in homologous recombination repair of double-strand DNA breaks. Together with BRCA1, proteins such as ATM, CHEK2, Tp53, BARD1 and BRIP1 are involved in the recognition of double-strand DNA breaks and the assembly of the repair proteins to the breakpoint (20–22). Once at the site, it is the function of the MRN complex to resect the blunt ends of the break and stabilize the break (23). At this point, with the assistance of PALB2, BRCA2 and RAD51 loading, strand invasion commences to complete the repair process (24). The question is whether the implementation of multigene panel testing could contribute to and complement the vision of better health systems for Africa across the continuum of cancer care. We believe that bridging the clinical implementation gap between research and service delivery (16, 25) will advance the knowledge of hereditary breast and ovarian cancer in South Africa as a representative of the African continent. Complementing targeted multigene panel testing with comprehensive genome-scale screening using WES/WGS offers unique learning and capacity-building opportunities. By prioritizing the development of resources needed to integrate research and service delivery, loss of employment opportunities can be prevented, while the scope of clinical research avenues available to African scientists are increased. We envisage a future where our translational research focus will encourage both public and private practicing healthcare practitioners to refer patients for genomic testing at the National Health Laboratory Service in South Africa.

## Materials and methods

### Study population

Blood samples of 414 patients attending various genetic clinics across South Africa were received at the National Health Laboratory Service Human Genetics laboratory in Bloemfontein, South Africa. The patients represented individuals diagnosed mostly with either breast or ovarian cancer, who requested diagnostic screening using NGS of genes involved in the development of hereditary breast and ovarian cancer. All the patients underwent pre- and post-test counseling at their respective referring institute, where clinical information together with disclosure of a personal and/or familial aggregation of cancer cases was provided by the referring physician and/or genetic counselor. The request was accompanied by written informed consent for DNA testing and data sharing. Patient details were de-identified for publication purposes. The cohort included mostly patients without a family history of cancer, diagnosed at an early age of onset (<45 years). Once analyzed, 95 patients were excluded from this study due to the absence of consent for data sharing. Race/ethnicity was self-reported and represented all main South African population groups. The data sets for the 15 genes are publicly available (ClinVar submission number SUB11378633 [SCV002504693–SCV002505346]).

### Targeted sequencing

DNA was extracted from peripheral blood using an adaption of a standard salting-out method (26). Germline DNA samples were screened using the NGS Oncomine™ BRCA Expanded Research Assay (Life Technologies, Carlsbad, CA, USA). The primer pools targeted the entire coding region and splice-site junctions. Multiplexed primer pools were used to construct the amplicon library using PCR-based targeted amplification, where after it was sequenced using the Ion GeneStudio™ S5 system (Life Technologies, Carlsbad, CA, USA). Raw signal data were analyzed using the Torrent Suite™ version 5.12.1 (Thermo Fisher Scientific Inc., Waltham, MA, USA). The pipeline included signaling processing, base calling, quality score assignment, trimming of the adapters (average read length 121 bps), read alignment, and quality control of mapping quality. The Ion Reporter™ Software version 5.18.2 (Thermo Fisher Scientific Inc., Waltham, MA, USA) was used for filtering out possible artifacts and the annotation of variants. Coverage analysis and variant calling were generated using the Torrent Variant Caller plugin software in the Torrent Server. The average coverage depths obtained were 936X (range 745–1164X).

Using the depth per read, quartile statistics was applied to calculate the average depth distribution around the mean across the samples. Samples that were located within the 2nd and 3rd quartile, were selected to construct a copy number variant baseline with the Ion Reporter CNV VCIB 4.0.0.1 algorithm. Copy number variant detection for the 15 genes was performed using an algorithm that normalized depth coverage across amplicons to predict the copy number or ploidy states. The computed baseline included a minimum of 100 control samples (each with an average of 70–140 million bases called greater than Q20 and a read count of 0.6–1.2 million), using regions with known ploidy states (https://assets.thermofisher.com/TFS-Assets/LSG/brochures/CNV-Detection-by-Ion.pdf).

### Multiplex ligation-dependent probe amplification

Multiplex ligation-dependent probe amplification was used to confirm copy number variants situated in genes for which pre-designed assays were readily available (MRC-Holland, Amsterdam, The Netherlands). These assays covered the complete coding regions for both *BRCA1* and *BRCA2*, whereas the design focused on well-established copy number variant regions or hot spots for *TP53*, *ATM*, *FANCD2*, *PALB2*, *CHEK2*, *BRIP1*, *BARD1*, and *NBN*. For less familiar genes, copy number variants detected using NGS were investigated and confirmed by comparing results for duplicated samples in separate runs in the presence of a positive copy number variant control (a quality indicator for library preparation). The ligated products were run together with a size standard on an ABI 3500XL Genetic analyzer (Applied Biosystems, Carlsbad, California, USA). Multiplex-ligation-dependent probe amplification data were analyzed using GeneMarker® software version 3.0.1 (SoftGenetics, LCC, State College, PA, USA). Confirmed copy number variants were named according to the Human Genome Variation Society (http://www.HGVS.org/varnomen) guidelines and classified using the adapted recommendations of the American Society of Medical Genetics and Genomics for the interpretation and reporting of a single-gene copy number variant (27). On occasion and depending on quality parameters, potential likely pathogenic and pathogenic variants (especially single nucleotide variants in homopolymer regions) were confirmed using Sanger DNA sequencing. Sanger sequencing was performed using the ABI Prism BigDye^®^ Terminator v3.1 cycle sequencing kit (Life Technologies, Carlsbad, CA, USA) according to the manufacturer’s protocols.

### Whole exome sequencing

Exploratory WES was performed for a limited number of *BRCA*-negative patients (*n*=16) and control samples of known genotype (n=4) to assist with the validation process of the BRCA Expanded 15-gene panel. Comparison of the data produced using the two technologies provided insight regarding the cost-benefit of WES for variant and gene discovery based on a virtual panel of 84 cancer-related genes. Simultaneously, potential analytical challenges were highlighted regarding optimal depth needed for accurate variant calling through coverage and variant spectrum. WES was performed according to the methods previously described as part of postgraduate training (10, 28). Genomic quality scores were determined on the LabChip^®^ GXII Touch (PerkinElmer, Waltham, MA, USA), using the DNA Extended Range Chip and genomic DNA Reagent Kit (PerkinElmer). The Ion AmpliSeq™ Exome RDY Kit was used to prepare exome libraries, where after these targets were amplified using the Ion AmpliSeq™ Exome RDY Panel and the Ion AmpliSeq Library Kit Plus. The products were combined, and primer sequences digested to generate barcoded libraries after purification with Agencourt™ AMPure XP magnetic beads. The barcoded exome libraries were combined for sequencing template preparation using the Ion 540 Chef Kit, where after the pooled library was loaded onto the Ion Chef for template preparation and enrichment using Ion 540 Chef Reagents. Sequencing was performed on the Ion Torrent Ion S5 platform and the Ion Reporter software used for variant calling (28) of high to moderate-risk cancer genes using the Invitae multi-cancer panel (**Table S1**, https://www.ncbi.nlm.nih.gov/gtr/tests/528909/).

### Variant classification

The clinical significance of single nucleotide variants was determined based on the framework of guidelines proposed by the American Society of Medical Genetics and Genomics and the Association for Molecular Pathology (29), including the amended versions designed specifically for *TP53* (30) and *ATM* (31). These guidelines together with freely accessible public databases such as ClinVar, LOVD, BRCA Exchange, and the genomic search engine VarSome (32) were used for ultimate variant classification. The mutation nomenclature was used according to Human Genome Variation Society recommendations. The genes analyzed were numbered and annotated using the National Center for Biotechnology Information chromosome and transcript reference sequences, as listed in **Tables 1**, **S1**. Variants detected were categorized according to the Evidence-based Network for the Interpretation of Germline Mutant Alleles consortium.

**Table 1:** Summary of variants detected during comprehensive screening of 319 patients using the Oncomine™ BRCA Expanded panel.

Gene	Transcript	mRNA length	Total *n* of variants	*n* and (%) of novel variants	% of intronic variants	% of coding variants	% of missense variants	% of synonymous variant	% of stop-gain variants	% of in- frame deletions	% of frameshift variants	*n* and (%) of actionable variants
ATM*	NM 000051.4	12,915 nt	155	16 (10.3)	48	52	69	26	I	I	0	2 *(* 1.3)
BARD*	NM 000465.4	5,478 nt	55	3 (5.5)	38	62	73	17	3	3	3	2 (3.6)
BRCA1*	NM 007294.4	7,088 nt	80	9 (11.3)	27	73	59	25	4	4	9	11 (13.8)
BRCA2*	NM 000059.4	1 1,954 nt	120	9 (7.5)	22	78	55	29	6	3	7	20 (16.7)
BRIP1*	NM 032043.3	8,182 nt	39	4 (10.3)	64	36	71	29	0	0	0	2 (5. 1)
*CDK12*	NM 016507.4	8,287 nt	31	3 (9.7)	39	61	44	56	0	0	0	0 (0)
CHEK2*	NM 007194.4	1,844 nt	20	1 (5.0)	43	57	40	40	10	0	10	4 (20.0)
*FANCD2*	NM 033084.6	4,879 nt	74	7 (9.5)	55	45	39	61	0	0	0	I (1.4)
PALB2*	NM 024675.4	4,008 nt	37	4 (10.8)	53	47	71	24	0	0	6	I (2.7)
*PPP2R2A*	NM 002717.4	3,923 nt	16	1 (6.3)	71	29	0	100	0	0	0	0 (0)
*MRE11*	NM 005591.4	6,841 nt	35	3 (8.6)	56	44	69	31	0	0	0	0 (0)
NBN*	NM 002485.5	4,622 nt	45	3 (6.7)	54	46	59	41	0	0	0	0 (0)
*RAD51B*	NM 001321818.2	1,273 nt	33	3 (9.1)	61	39	67	33	0	0	0	0 (0)
*RAD54L*	NM 003579.4	3,078 nt	35	2 (5.7)	40	60	58	37	0	5	0	0 (0)
TP53*	NM 000546.6	2,512 nt	29	2 (6.9)	65	35	44	44	0	0	1 1	1 (3.5)
*Total*			804	70 (8.7)	45	55	59	33	3	2	4	46 (5.7)

*n*, number; *genes included in the latest National Comprehensive Cancer Network (NCCN guidelines, version 2.2022). Variants observed in the untranslated regions, together with splice-site variants are included as intronic variants.

## Results

NGS was used to determine the presence and frequency of likely- to pathogenic variants in genes responsible for hereditary breast and ovarian cancer syndrome in South Africa. The 15-gene panel was designed as a successor to the Oncomine™ BRCA Research Assay (Life Technologies, Carlsbad, CA, USA) (33) (**Table 1**) previously used in a subset of our study population to identify defects in genes involved in the homologous recombination DNA damage repair pathway, which affect response to DNA-damaging therapies (34, 35). While patients with pathogenic variants in any of the 15 genes included in the NGS panel can be expected to benefit from poly (ADP-ribose) inhibitor treatment, this therapy is not available in the public sector and our findings served to identify a need for targeted therapy as validation of the assay would also have clinical implications for at-risk family members in the broader community. The validation of the expanded panel was simplified as the two assays shared identical primer pools for *BRCA1* and *BRCA2*. Using the existing *BRCA1/2* variant data [compiled from the two-gene *BRCA1/2* assay (*n*=1 089), together with the research-based WES results (*n*=20)], a total of 344 *BRCA1/2* variants representing both single- and copy number variants were available for cross-correlation (5).

Our aim was to assess the overall prevalence of potentially relevant cancer risk gene variants in the samples received between June 2021 and February 2022 (*n=*414 patients), using multigene panel testing. For the section of the cohort with clinical data and consent for data sharing (*n*=319), a total of 48 actionable variants were identified (**Table 2**). *BRCA1* and *BRCA2* contributed the most to the pool of likely- to pathogenic variants recorded (*n*=34/48, 69.4%) (**Table 2**), with the majority observed for *BRCA2* (*n=*32/48, 66.7%). Five of the six South African specific founder variants were detected, two in *BRCA1* (*n*=2/48; c.1374delC, p.Asp458GlufsTer17 and c.2641G>T,p.Glu881Ter) and three in *BRCA2* (*n*=10/48; c.582G>A,p.Trp194Ter; c.5771_5774delTTCA, p.Ile1924ArgfsTer38 and c.7934del, p.Arg2654AsnfsTer3) (**Table S2**) (5, 6, 8, 36, 37). The expanded panel detected 10 and 12 non-founder pathogenic variants in *BRCA1* and *BRCA2*, respectively, together with three *BRCA1* copy number variants all representing single exon deletions, confirmed using multiplex ligation-dependent probe amplification (**Table S2**). The search revealed three novel variants, two in *BRCA1* with a single in *BRCA2* (*BRCA1* c.302_303delATinsGA, *BRCA1* c.125dup and *BRCA2* c.1705C>T, p.Gln569Ter) (**Table S2** **)**. *BRCA1* c.302_303delATinsGA represented a new splice-site variant, with *BRCA1* c.125dup (situated in the critical zinc finger domain), postulated to negatively influence the binding between BRCA1 and BARD1. As three separate null variants at the same position as *BRCA2* c.1705C>T, p.Gln569Ter were previously reported as pathogenic by an expert panel in ClinVar (c.1705del, p.Gln569ArgfsTer4, c.1705insG, p.Gln569AlafsTer21 and c.1705_1706del, p.Gln569GlufsTer20), the variant was classified as likely pathogenic based on the same criteria. Most of the variants were patient-specific (**Table S2**).

**Table 2:** Testing results by gene category and personal characteristics.

		Results by gene category, number (%) of LP and PVs		
	Actionable cases by test result, number (%)	High-risk *BRCA1* & *BRCA2*	Non-*BRCA* high-risk BC and/or OVC genes	Moderate to lower risk BC and/or OVC genes
**Characteristic**				
*Type of cancer diagnosed at any age*				
BC (*n=*287)	41* (13.6)	27 (9.4)	2 (0.7)	12 (4.2)
OVC (*n=*3)	1 (33.3)	1 (33.3)	0 (0)	0 (0)
BC & another ca (*n=*15)	3 (20.0)	2 (13.3)	0 (0)	1 (6.6)
Prostate ca (*n=*9)	1 (11.1)	1 (11.1)	0 (0)	0 (0)
Other ca types (*n=*5)	1 (20.0)	1 (20.0)	0 (0)	0 (0)
*Gender*				
Female (*n=*298)	44* (14.1)	30 (10.6)	2 (0.7)	12 (4.0)
Male (*n=*21)	3 (14.3)	2 (9.5)	0 (0)	1 (4.8)
*Ethnicity*				
African (*n=*188)	27 (14.7)	18 (9.6)	1 (0.5)	7 (3.7)
Caucasian (*n=*36)	10 (27.7)	5 (13.8)	0 (0)	4 (11.1)
Mixed ancestry (*n=*51)	7 (13.7)	6 (11.8)	1 (1.9)	0 (0)
Asian (*n=*32)	5 (15.6)	3 (9.3)	0 (0)	2 (6.2)
Non-SA (*n=*12)	0 (0)	0 (0)	0 (0)	0 (0)
*Age at onset*				
Diagnosis < 45 years (*n=*168)	26 (15.5)	18 (10.7)	1 (0.6)	7 (4.2)
Diagnosis ≥ 45 years (*n=*151)	23 (15.2)	16 (10.6)	0 (0)	7 (4.6)
Total (*n=*319)	49* (14.7)	34* (10.0)	1 (0.3)	14 (4.3)

BC, breast cancer; OVC, ovarian cancer; ca, cancer; LP, likely pathogenic; PV, pathogenic variant; SA, South African; *two female BC patients were double heterozygotes with PVs in both *BRCA1* and *BRCA2*. Percentages are calculated as the total actionable cases per characteristic.

Apart from *BRCA1* and *BRCA2, ATM* (*n*=4/48), *CHEK2* (*n*=3/48) and *BARD1* (*n*=2/48) contributed the most to the positive variant detection rate, with single variants observed for *BRIP1*, *PALB2* and *TP53* (**Table S2**). The majority represented single nucleotide variants, with only a single copy number variant identified for *ATM* (**Table S2** and **Figure S1**). *ATM* was the largest non-*BRCA* gene contributing to the increased mutation positivity rate (8.3%, 4/48), followed by *CHEK2* (3/48). All the variants observed for these two genes were detected in African patients. One of the variants, *CHEK2* c.283C>T, p.Arg95Ter (rs587781269), represented a recurrent variant observed in two patients, together with a single occurrence reported in an earlier South African study (**Table S2**) (38). Functional assays confirmed the protein to be non-functional in terms of kinase activity and dimerization (39). A multicentre association study involving more than 113 000 women associated truncating variants in *CHEK2* with an increase in breast cancer risk [odds ratio of 2.13 (1.60–2.84)] furthermore influenced by the ER status of the tumor [ER+ tumors 2.67 (95% CI, 2.3–3.11)] (9). This accounted in part for the early age of breast cancer diagnosis in a young male diagnosed with an ER-positive tumor. Although preliminary, this variant could represent the first non-*BRCA* recurrent pathogenic variant observed in the South African population.

The remainder of variants was observed for *PALB2*, *TP53*, *BARD1*, and *BRIP1*, with a single likely- to pathogenic variant observed for all genes except *BARD1* (*n*=2) (**Table S2**). The *PALB2* c.3507_3508del, p.His1170PhefsTer19 variant resulted in the extension of the WD40 domain that plays a crucial role in the function and stability of the protein during its interaction with BRCA2 (40). The complex rearrangement observed for *TP53* (c.158_163delGGTTCAinsAT, p.Trp53SerfsTer69) impacted various critical domains of the protein including the interaction with CCAR2 and HRMT1L2 (41). As the p53 protein is considered the guardian of the genome, this null variant located in such a hotspot area is associated with a predisposition to cancer in individuals with Li-Fraumeni syndrome (42). A complete list of the VUSes detected is presented in **Table S3**.

In total, 804 filtered variants were observed distributed amongst the various genes, with 51.5% (414/804) occurring in the coding regions (**Table 1**). The bulk of these variants were heterozygous missense variants (59%). Four of the genes had a novel variant percentage above 10% (*ATM*, *BRCA1*, *BRIP1*, and *PALB2* (32/53)) situated in a domain or important region. *BRCA1* and *BRCA2* contributed the most nonsynonymous and frameshift variants to the mutation-positive variant pool. Grouping variants based on clinical significance resulted in 46% (370/804) classified as benign; 41% (330/804) as likely benign; 9% (73/804) as a VUS; 0.1% (1/804) as likely pathogenic and 5.7% as (46/804) pathogenic (**Table 1**). The initial VUS percentage was higher, largely due to the inclusion of intronic variants located further than 25 bases from the splice site. These were ultimately reclassified as likely benign based on predicted consequences (**Table S3** **)**.

The majority of patients were African (*n*=197, 61.8%), with the remaining 38.2% of the cohort comprising of Asian (*n*=32, 10.0%, all South African Indian), Caucasian (*n*=36, 11.3%, mostly Afrikaner) and the Mixed ancestry population (*n*=51, 16.0%) (**Table 2**). Ethnicity was not indicated for three patients. The mean age of diagnosis was 44.8 years, with most presenting with breast cancer (*n*=302, 94.7%). The additional cancer types included ovarian, cervical, womb, thyroid, prostate, rectal, pancreatic, colon and skin cancer (*n*=31, 9.7%). A significant number of patients (*n*=166, 52.0%) reported a family history of cancer, although in most cases represented by only a single affected family member. The presence of a family history was omitted for 42 patients and included patients that were adopted as a child (13.1%). Most of the patients presented with stage II breast cancer (*n*=96, 30.1%) (**Table S3**).

**Figure 1:**
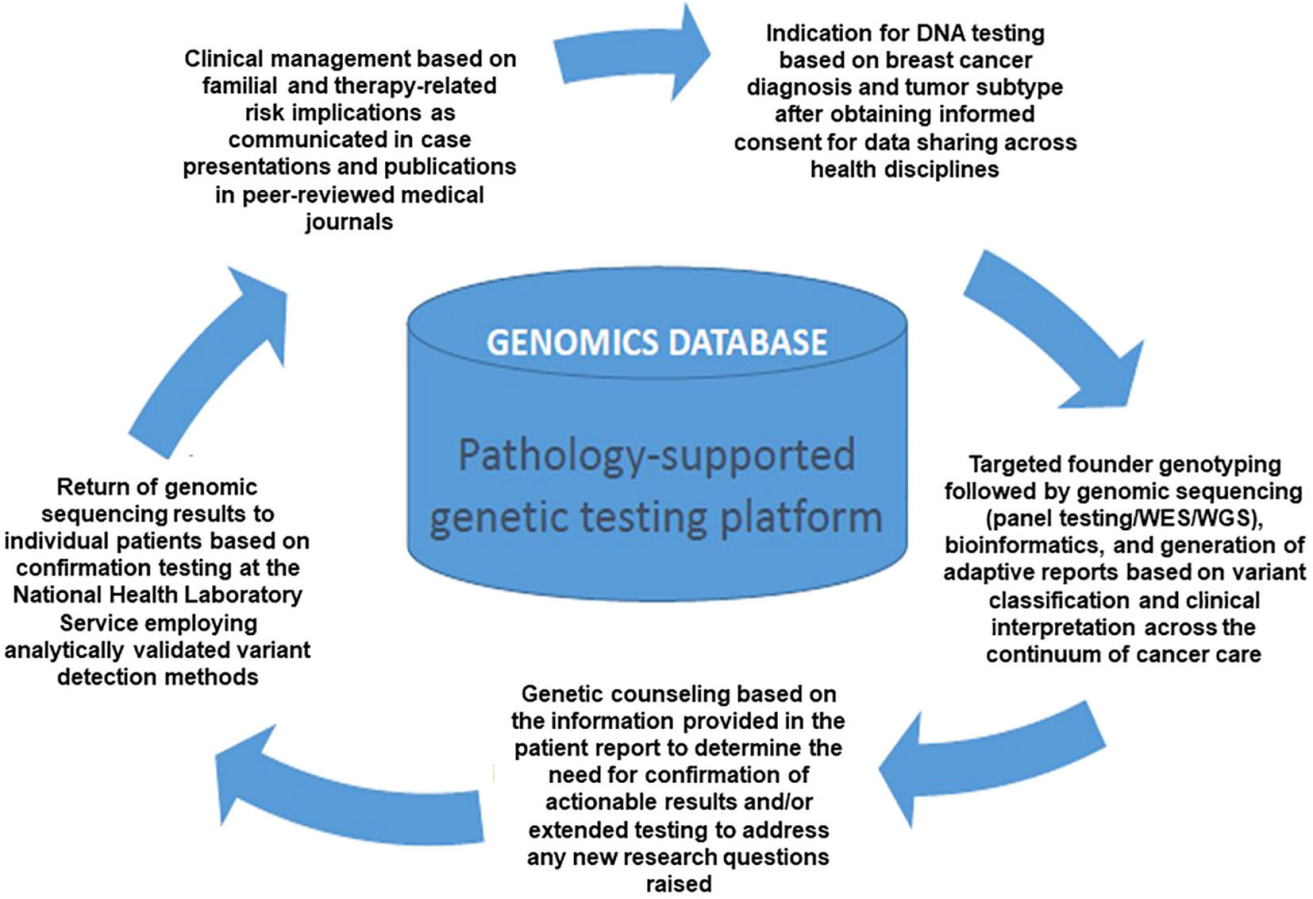
Flow diagram illustrating integrating research into diagnostic service *via* a pathology-supported genetic testing platform, developed as a cost saving strategy in South Africa (Adapted with permission from Baatjes et al. [43)].

Twenty patients were selected for WES based on their tumour type and family history of breast/ovarian cancer, including four controls of known genotype replicated in this study. Actionable pathogenic variants were detected in four of the 16 genetically uncharacterized cases, including a pathogenic *CHEK2* c.283C>T p.Arg95Ter terminating variant in two patients (**Table S2**). Truncating variants were also detected in two lesser-known cancer susceptibility genes, namely *PTCH1* (NM_000264.5):c.4187delG, p.Gly1396AspfsTer56 and *KIT* (NM_000222.3):c.930delA, p.Gly311AspfsTer8. Since WES was performed in a research environment, DNA samples of the two patients with *CHEK2* c.283C>T p.Arg95Ter were re-tested using the multigene *BRCA* expanded panel to confirm the result. This enabled the translation of research findings into clinical practice *via* the respective referring doctors and patients with genetic counselling support, as illustrated in **Figure 1**. Except for a *PALB2* variant with conflicting evidence of pathogenicity (data not shown) investigated further, WES analysis using a virtual panel of 84 cancer-related genes did not reveal any actionable mutations in the 12 unresolved breast cancer patients.

## Discussion

In this study we combined targeted and exploratory NGS technologies to develop a comprehensive research-driven genetic testing service for breast and ovarian cancer. This approach has unlimited potential regarding the number of genes accessible for application of personalized genomic medicine. In contrast to targeted NGS, WES is not restricted by the number of genes that can be analysed and this benefit was evident from detection of two novel variants (*PTCH1* c.4187delG and *KIT* c.930delA) in genes not included in the 15-gene Oncomine™ BRCA Expanded Panel. WES includes all the coding regions of the human genome containing approximately 85% of clinically relevant gene variants, but data coverage is not uniform. Therefore, virtual NGS panels applied to WES data WES data in a research environment is ideally suited to support continuous updating of the risk categories being allocated to cancer susceptibility genes and overlapping or distinct pharmacogenetic markers. All the patients with likely- to pathogenic variants detected with the 15-gene panel are eligible for targeted therapy and implementation of prevention strategies. By knowing the risks, affected patients and their at-risk family members who may benefit from clinical interventions can be identified and referred to clinicians for implementation of appropriate treatment strategies. *BRCA1* and *BRCA2* contributed the most toward the percentage of actionable variants identified, with the majority representing *BRCA2*. Apart from the founder *BRCA1/2* variants contributing 35.3% [12/34] to the positivity rate, the 15-gene panel revealed seven variants identified for the first time in South Africa, despite more than 1 400 patients comprehensively screened thus far in our laboratory. This finding illustrates the unique genetic architecture of the South African population and highlights the underrepresentation of African variants in international databases. Moreover, with the necessary approvals being applied for as part of postgraduate studies, we may in future extend the number of overlapping NGS data sets stored in the existing cancer genomics database generated though parallel testing of the 15-gene panel and WES/WGS.

Comparison of our results with those reported by the BCAC highlighted the effectiveness of established selection criteria for familial genetic risk screening, as the average age at onset ranged between 40–55 years of age, similar to our patients (72% diagnosed before age 50 years) (9) (**Supplementary Data**). Appropriate selection of patients for genetic investigations was reflected by the expected genes and variant types in which pathogenic variants were identified. Should lower-risk patients diagnosed at a later age be representative of most cases in the cohort, a potentially different spectrum of genes and pathogenic variants might be observed. Currently, the lesser-known genes included in the 15-gene panel lack odd ratios and are problematic for quantifying cumulative risk without an appropriate control cohort based on healthy older women. Our data reflect the progress made in South African policies relating to patient selection criteria for the identification of high penetrant variants. At the same time, it also accentuates the inequalities that remain regarding genetic testing of the broader population. This limits our understanding of the healthy human genome and remaining hurdles to overcome for patients to truly benefit from genetic testing. Since research translation requires a seamless process from sample collection to report generation, the development of clinical decision support materials as an integral part of implementation studies is important. Towards this goal, we summarised the current lifetime or absolute risk estimates for genes containing clinically relevant variants in the South African study cohort, as shown in Supplementary **Table S4**. The clinical guidelines indicating the risks and odds ratios for each of the genes, as well as proposed management and potential therapeutic options for mutation carriers, were based on current literature and NCCN Guidelines considered useful for inclusion in patient reports in future.

Genetic testing strategies currently available in the public sector of South Africa do not routinely include WES/WGS or use of genome wide genotyping arrays. While advances in NGS technologies have quickly highlighted WES/WGS as the ultimate discovery tool to gain insight into an individual’s genetic code (44), it remains costly. Therefore, we use WES/WGS in skills development programs to ultimately curb an increasing trend among healthcare practitioners to refer patient samples abroad through outsourcing. WES-related research is not only an attempt to build and expand the diagnostic service in-house for South African patients, but also to ensure that diagnostic and research material/data remain in the country as far as possible. The safekeeping of valuable data forms the basis for teaching and training of the next generation of South African medical practitioners, including medical scientists and genetic specialists. Storage of genetic information together with phenotypical observations unique to the local population and individual patients, will over time enable retrospective genotype-phenotype association and follow-up studies. This pathology-supported genetic testing approach accelerates data collection and has the potential to reduce the anxiety for patients and their families relating to the odyssey of health research investigations that end up in VUSes or variants discovered in genes with insufficient clinical evidence to act on. This was clearly demonstrated by Moremi et al. (11), with return of research results to family members of deceased breast cancer patients with somatic *TP53* variants. Germline WES of stored DNA samples excluded the risk of Li-Fraumeni syndrome for the patients’ family members. As the Department of Health is currently not in a position to offer WES/WGS to patients in the diagnostic laboratory, our data-sharing platform is accessible across participating healthcare institutions to assist with investigations in selected patients. **Figure 1** illustrates this process, which was first described by Baatjes et al. (43) as a further inventive step to identify pathogenic variants that were previously missed, again highlighting the need for universal *BRCA1/2* screening supported by the unique genetic structure of the South African population.

In addition to the detection of a pathogenic *CHEK2* variant in two cases with use of the 84-gene virtual WES panel, actionable variants were also detected in the lesser-known cancer-associated genes *PTCH1* and *KIT*. *PTCH* is involved in the hereditary condition Nevoid basal cell carcinoma syndrome (NBCCS) or Gorlin syndrome (45, 46). The gene has an autosomal dominant inheritance pattern with nearly complete penetrance and variable expressivity (47). PTCH1 is a transmembrane glycoprotein that is composed of 12 transmembrane domains. The syndrome is characterized by developmental abnormalities and tumor predisposition, with a diagnosis based on the occurrence of multiple basal cell carcinomas, palmar and plantar epidermal pits (48) and/or jaw keratocysts (48, 49). This gene is also associated with ovarian fibromas diagnosed at a mean age of 30 years and potentially acts as a tumor suppressor gene (47), with many germline pathogenic variants since described in the literature. The *KIT* gene encodes for a protein located in the cell membrane of certain cell types and provides instructions to produce receptor tyrosine kinases, which are responsible for signal transmission from the cell surface into the cell through signal transduction. The KIT protein is activated by the binding of a growth factor, namely the stem cell factor. Together, they regulate a variety of crucial cellular processes such as proliferation and differentiation, cell survival and cell cycle control (50, 51). The aberrant activation of KIT results in the deregulation of the signaling networks which has been associated with the progression of many cancer types such as melanoma, gastrointestinal stromal tumor, and stomach cancers (52–54).

**Figure 2:**
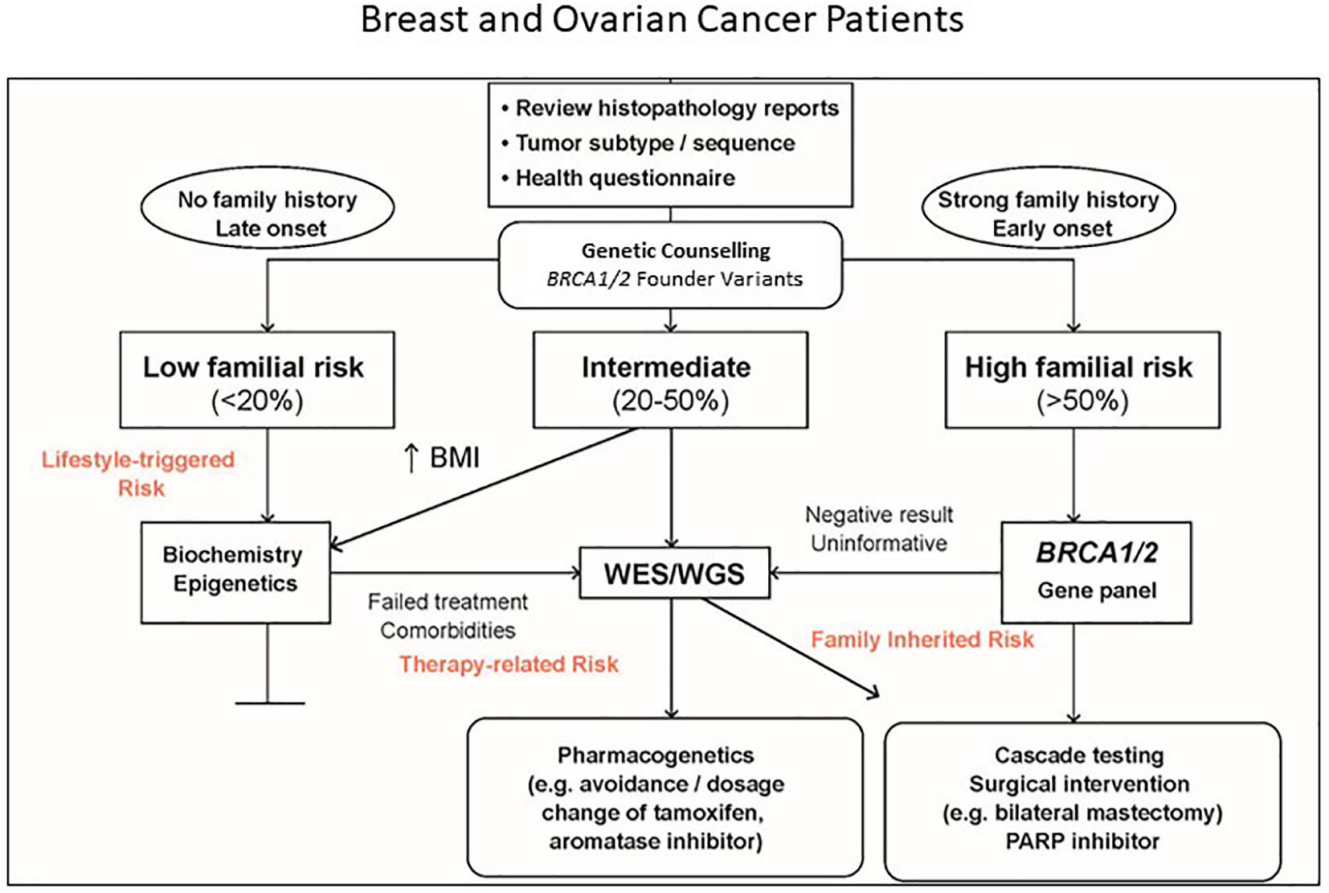
Pathology-supported genetic testing algorithm extending from *BRCA1/2* founder variant testing to whole exome/genome sequencing (WES/WGS) in unresolved cases following targeted multigene panel testing. BRCA Expanded gene panel gene panel is used in *BRCA1/2* founder variant-negative cases with a high familial risk profile based on early age at diagnosis and/or a strong family history of cancer. Clinical assessments such as body mass index (BMI) and relevant biochemical testing may be considered in genetically uncharacterised cases to assist with lifestyle-triggered and therapy-associated risk stratification (Modified with permission from Mampunye et al. (16).

The expansion of routine genetic testing from conventional *BRCA1/2* testing to multigene testing led to a relatively high diagnostic yield of 15%, with approximately one-third of positive cases (12/34, 35.3%) found to be known South African founder variants. Although no official cost-effectiveness analysis has been performed in South Africa, these results justify the incorporation of *BRCA1/2* founder variant testing in the screening algorithm recommended by Mampunye et al. (16). Since transcriptional gene profiling routinely used in the private sector to prevent chemotherapy overtreatment in patients with early-stage hormone receptor-positive breast cancer is not available in the state sector, we modified the screening algorithm by omitting the assessment of RNA extracted from formalin-fixed paraffin embedded tumor biopsies. This is shown in **Figure 2**, starting with *BRCA1/2* founder variant testing extended to BRCA-expanded gene panel testing in high-risk cases based on the age at diagnosis and a strong family history of cancer. These clinical indicators form part of the WES pre-screen algorithm previously described by van der Merwe et al. (10) for risk stratification when considering prophylactic surgery or magnetic resonance imaging. With identification of low-to-moderate penetrance genes, clinical assessments such as body mass index (BMI) may help to inform intervention strategies targeted at gene–environment interaction to reduce recurrence risk. For unresolved familial cases and patients with treatment failure or medication side effects, proceeding to WES/WGS may be beneficial beyond a singular health concern or research objective.

While electronic gatekeeping regarding requirements for genetic testing has been implemented by the Department of Health to ensure that referring clinicians adhere to the new national guidelines, use of the newly designed point-of-care (POC) assay (8, 16) as a first-tier test for all breast and ovarian cancer patients has the potential to not only reduce cancer incidence but also improve survival by identifying high-risk variants in patients in need of risk-reducing treatments. The rapid POC assay is inexpensive (estimated purchase price ZAR 1 000) and can be used for all breast and ovarian cancer patients, irrespective of the Manchester score, ethnicity, tumour subtype or a history of cancer in other family members. The integrated workflow from sample collection to nucleic acid sequence analysis makes it ideal for targeted genetic testing in any primary/rural health clinic with access to online genetic/genomic counseling support and genotype data export for independent expert review. Sample collection using buccal swabs for POC DNA testing could replace the more expensive (ZAR 2 508.54) laboratory-based founder variant detection assay once fully validated against confirmed positive and negative controls. Such a rapid screen may increase access into the right care pathway as the average South African female has very limited knowledge regarding potential risk factors, although most are aware that a breast or lump in the armpit could be a sign of disease (55, 56). As both the two-gene assay and the multigene test are currently offered at the same price (ZAR 7 912.86), the expanded test will be more beneficial for patients, even though costly poly (ADP-ribose) inhibitors are not yet available to mutation carriers in the public sector.

Early detection is of the utmost importance, since South African patients have no or very limited access to specialized diagnostic procedures and targeted therapies such a Herceptin (ZAR 20 000 for a single dose). Scans and imaging are major cost drivers, ranging from ZAR 1 800 for a mammogram to ZAR 3 000 – 15 000 for MRI and CT scans (medical aid tariffs) (57). Regarding treatment, the costs for one episode of chemotherapy were calculated to be approximately ZAR 16 259 in the absence of any adverse events (58). This drastically increased to ZAR 36 465 for patients who developed neutropenia, the most common adverse event encountered during their study. Surgery as a primary treatment option ranged from ZAR 30 000 for a lumpectomy to ZAR 150 000 – 250 000 (according to medical aid tariffs - https://www.1life.co.za/blog/cost-treat-breast-cancer) for bilateral mastectomies that include reconstruction. The costs indicated were not compared to other countries especially on the African continent, due to population differences, varying cancer incidence, and different health funding systems. Based on a cost-effectiveness analysis performed in the United States of America and the United Kingdom regarding the implementation of unselected multigene testing for all (59), it may be advisable to permit high-risk multigene genetic testing for all patients affected with breast or ovarian cancer in South Africa. Currently, multigene panel testing for hereditary breast cancer internationally delivers on average a prevalence of 1-14% positivity in the non-*BRCA* genes but simultaneously is accompanied by a high level of VUSes (60). This is likely to eventually incur higher costs over time when evaluated for its benefits and may increase the anxiety of patients.

This study was limited by the number of genes studied using the 15-gene NGS panel; hence the parallel use of WES rendered sustainable through research grants and postgraduate student training that open many benefit-sharing opportunities (61). The challenges associated with selection of the most appropriate genes for panel testing may be addressed by an adaptive reporting system currently developed as part of WES/WGS research. More information is needed about the prevalence, penetrance, and increased risks associated with the variant spectrum identified in the study cohort. Multi-gene panel testing is not yet ready for non-specialized clinical use without clear guidelines as the identification of actional variants in the more unfamiliar genes such as *CDK12* and *MRE11*, remains a problem. As 13/319 of the breast cancer cases analyzed in this series were lobular carcinomas (4.1%), likely- to pathogenic variants in the pleiotropic *CDH1* gene (62) would have been missed. Studies are underway to adjust the 15-gene panel to include this gene, requiring knowledge of tumor pathology and other genes for which consensus risks and management guidelines exist, to advance towards better health care for our patients.

## Conclusions

The elusive goal of selecting the right number and type of genes for breast and ovarian cancer pharmacodiagnostics can be achieved in future by application of pathology-supported genetic testing extending from *BRCA1/2* founder variant screening to multigene panel testing complemented by WES/WGS in genetically uninformative cases. The use of pathology to bring genetics into the treatment domain is in line with the framework for implementation of genomic medicine in Africa, substantiated by a readiness assessment (63) applied in this study focused on the cost implications reported. We conclude that multigene panel testing offers a viable improvement over *BRCA1/2*-only sequencing, by contributing to the positivity rate of actionable pathogenic variants in 15% of cases (48/319). This finding is in line with international data and confirmed the necessity for universal *BRCA1/2* founder variant screening extended to NGS in unresolved cases to help prevent morbidity and mortality. Despite major strides made over the past decade to catch up with first-world countries, uptake of genetic diagnostic services will not reach its full potential unless genomic literacy is increased. Only then will the new era of genomics truly have an impact on healthcare in Africa. It is the opinion of the authors to prioritize the screening of genes for which the results can be applied in clinical practice, while enriching the cancer genomics database through return of actionable WES/WGS results towards the implementation of a learning healthcare system.

## Contribution

Targeted and exploratory NGS technologies was combined in this study to develop a comprehensive research-driven genetic testing service for breast and ovarian cancer in South Africa. This approach has unlimited potential regarding the number of genes accessible for implementation of personalized genomic medicine. In contrast to targeted NGS, WES is not restricted by the number of genes that can be selected for inclusion in a virtual panel that can be analysed simultaneously. However, base coverage is not uniform throughout the human genome and therefore targeted gene panel testing is preferred in a diagnostic setting. Since all the patients with likely- to pathogenic variants detected with the use of a 15-gene panel are eligible for targeted therapies and implementation of prevention strategies, their at-risk family members may also benefit from knowledge of the familial risks with treatment implications. Implementation of the 15-gene panel resulted in the incorporation of genetic results into routine patient care, by providing more informative results for clinical decision-making at no additional costs. Utilization of a pathology-supported genetic testing platform for return of actionable WES results obtained in parallel to targeted NGS in unresolved cases, effectively bridged the clinical implementation gap between research and service delivery.

## Data availability statement

The datasets presented in this study can be found in online repositories. The names of the repository/repositories and accession number(s) can be found in the article/**Supplementary Material**.

## Ethics statement

The studies involving human participants were reviewed and approved by the Health Sciences Research Ethics Committee of the University of the Free State (UFS-HSD2021/0704/2202), South Africa. Provincial approval was also obtained from KwaZulu-Natal (KZ_202201_013), North-West Province (NW_202202_007) and the Western Cape (WC_202202_019) for the inclusion of their patients. Lastly, the National Health Laboratory Service permitted the use of the data (reference PR2110611). Translational research was performed under a collaborative material transfer and data sharing agreement (S006652) approved by Stellenbosch University, South Africa, including ethics reference numbers N09/08/224 and C19/06/020. The patients/participants provided their written informed consent to participate in this study.

## Author contributions

NM, HC and JO made substantial contributions to the conception, design and completion of this project. NM, HS, and KN obtained ethics approval. NM and KN selected the data for analysis and performed the genetic studies together with HS, JO, and MK. MK provided scientific insight following a gap analysis and applied for funding for research translation into clinical practice. All authors contributed to the article and approved the submitted version.

## Supplementary material

Supplementary Figure 1 Confirmation of the large deletion involving exons 62 and 63 in *ATM* detected with the Oncomine™ BRCA Expanded panel using next-generation sequencing and confirmed with multiplex ligation-dependent probe amplification. **(A)** Visualization report for the next-generation sequencing data using Ion Reporter™ Genomic Viewer (IRGV). The copy number data representing the various *ATM* exons are enclosed in the black box. The reduction in copy number for exons 62 and 63 are circled. **(B)** Graphical multiplex ligation-dependent probe amplification presentation confirming the deletion of *ATM* exons 62 and 63 using GeneMarker® software for SALSA® MLPA® P0190-D1.
