# Supplementary figures and images for: Implementation of multigene panel testing for breast and ovarian cancer in South Africa: A step towards excellence in oncology for the public sector
Source: Front Oncol. 2022 Dec 7;12:938561. doi: 10.3389/fonc.2022.938561 (PMC9768488; doi:10.3389/fonc.2022.938561)

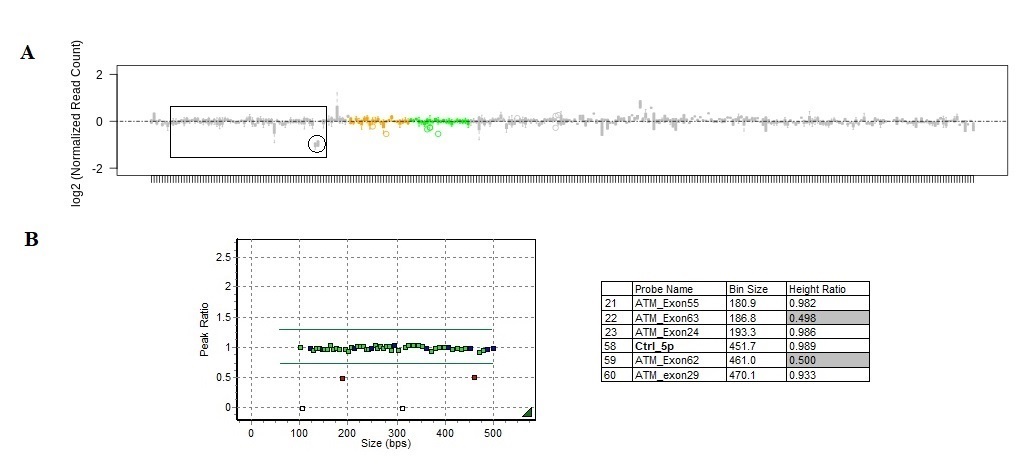

Supplement: Supplementary Figure 1 — Confirmation of the large deletion involving exons 62 and 63 in ATM detected with the Oncomine™ BRCA Expanded panel using next-generation sequencing and confirmed with multiplex ligation-dependent probe amplification. (A) Visualization report for the next-generation sequencing data using Ion Reporter™ Genomic Viewer (IRGV). The copy number data representing the various ATM exons are enclosed in the black box. The reduction in copy number for exons 62 and 63 are circled. (B) Graphical multiplex ligation-dependent probe amplification presentation confirming the deletion of ATM exons 62 and 63 using GeneMarker® software for SALSA® MLPA® P0190-D1. [file Image_1.jpg]
