# Supplementary material for: Implementation of multigene panel testing for breast and ovarian cancer in South Africa: A step towards excellence in oncology for the public sector
Source: Front Oncol. 2022 Dec 7;12:938561. doi: 10.3389/fonc.2022.938561 (PMC9768488; doi:10.3389/fonc.2022.938561)
Supplement: Supplementary file 2 [file Table_1.docx]

**SUPPLEMENTARY TABLE S1**: A virtual panel of 84 genes was screened using the Invitae^TM^ multi cancer gene list for the presence of clinically relevant variants in 20 breast and ovarian cancer patients using whole exome next-generation sequencing data. The genes indicated with an * are also included in the Oncomine^TM^ BRCA Expanded Research Assay (Life Technologies, Carlsbad, CA, USA) ThermoFisher which was used for multigene panel sequencing of the cohort.

| **Gene** | **Transcript** | **Gene** | **Transcript** | **Gene** | **Transcript** | **Gene** | **Transcript** |
| --- | --- | --- | --- | --- | --- | --- | --- |
| AIP | NM_003977.4 | CTNNA1 | NM_001903.5 | MUTYH | NM_001128425.2 | RUNX1 | NM_001754.4 |
| ALK | NM_004304.5 | DICER1 | NM_030621.4 | NBN* | NM_002485.5 | SDHA | NM_004168.4 |
| APC | NM_000038.6 | DIS3L2 | NM_152383.5 | NF1 | NM_001042492.3 | SDHAF2 | NM_017841.4 |
| ATM* | NM_000051.3 | EGFR | NM_005228.5 | NF2 | NM_000268.4 | SDHB | NM_003000.3 |
| AXIN2 | NM_004655.4 | EPCAM | NM_002354.3 | NTHL1 | NM_002528.7 | SDHC | NM_003001.5 |
| BAP1 | NM_004656.4 | FH | NM_000143.4 | PALB2* | NM_024675.4 | SDHD | NM_003002.3 |
| BARD1* | NM_000465.4 | FLCN | NM_144997.7 | PDGFRA | NM_006206.6 | SMAD4 | NM_005359.5 |
| BLM | NM_000057.4 | GATA2 | NM_032638.5 | PHOX2B | NM_003924.4 | SMARCA4 | NM_001128849.1 |
| BMPR1A | NM_004329.3 | GPC3 | NM_004484.3 | PMS2 | NM_000535.7 | SMARCB1 | NM_003073.3 |
| BRCA1* | NM_007294.4 | GREM1 | NM_013372.6 | POLD1 | NM_001256849.1 | SMARCE1 | NM_003079.5 |
| BRCA2* | NM_000059.3 | HOXB13 | NM_006361.6 | POLE | NM_006231.4 | STK11 | NM_000455.5 |
| BRIP1* | NM_032043.3 | HRAS | NM_005343.2 | POT1 | NM_015450.3 | SUFU | NM_016169.4 |
| CASR | NM_001178065.2 | KIT | NM_000222.3 | PRKAR1A | NM_212471.2 | TERC | NR_001566.1 |
| CDC73 | NM_024529.5 | MAX | NM_002382.5 | PTCH1 | NM_000264.5 | TERT | NM_198253.3 |
| CDH1 | NM_004360.5 | MEN1 | NM_000244.3 | PTEN | NM_000314.8 | TMEM127 | NM_017849.3 |
| CDK4 | NM_000075.3 | MET | NM_001127500.3 | RAD50 | NM_005732.4 | TP53* | NM_000546.5 |
| CDKN1B | NM_004064.4 | MITF | NM_000248.3 | RAD51C | NM_058216.3 | TSC1 | NM_000368.5 |
| CDKN1C | NM_000076.2 | MLH1 | NM_000249.3 | RAD51D | NM_133629.3 | TSC2 | NM_000548.5 |
| CDKN2A | NM_001195132.1 | MSH2 | NM_000251.3 | RB1 | NM_000321.2 | VHL | NM_000551.3 |
| CEBPA | NM_004364.4 | MSH3 | NM_002439.5 | RECQL4 | NM_004260.4 | WRN | NM_000553.6 |
| CHEK2* | NM_007194.3 | MSH6 | NM_000179.3 | RET | NM_020975.6 | WT1 | NM_024426.6 |
