# Supplementary material for: Implementation of multigene panel testing for breast and ovarian cancer in South Africa: A step towards excellence in oncology for the public sector
Source: Front Oncol. 2022 Dec 7;12:938561. doi: 10.3389/fonc.2022.938561 (PMC9768488; doi:10.3389/fonc.2022.938561)
Supplement: Supplementary file 3 [file Table_2.docx]

**TABLE S2:** Actionable variants (likely- to pathogenic) identified for the South African cohort using the Oncomine BRCA Expanded panel. Established South African founder variants are highlighted.

| **Variant** | | **Protein** | | **Cancer type in**  **index or family** | | **Exon** | | *n* **of patients with variant** | | **dbSNP/dbVar**  **number** | | | |
| --- | --- | --- | --- | --- | --- | --- | --- | --- | --- | --- | --- | --- | --- |
| ***BRCA1*** | | | | | | | | | | | | |  |
| NM_007294.4(BRCA1): c.68_69delAG | NP_009225.1: p.Glu23ValfsTer17 | | BC | | 2 | | 1 | | rs80357914 | | |  |  |
| NM_007294.4(BRCA1): c.125dup | NP_009225.1: p.Phe43IlefsTer23 | | BC, & OVC | | 3 | | 1 | | novel | | |  |  |
| NM_007294.4(BRCA1): c.191G>A | NP_009225.1: p.Cys64Tyr | | BC, gastric ca, & colon ca | | 4 | | 1 | | rs55851803 | | |  |  |
| NM_007294.4(BRCA1): c.302_303delATinsGA | NP_009225.1: p.Tyr101Ter | | BC | | 6 | | 1 | | novel | | |  |  |
| **NM_007294.4(BRCA1): c.1374delC** | **NP_009225.1: p.Asp458GlufsTer17** | | **BC** | | **10** | | **1** | | **rs397508862** | | |  |  |
| NM_007294.4(BRCA1): c.1603G>T | NP_009225.1: p.Gly535Ter | | BC | | 10 | | 1 | | no rs | | |  |  |
| NM_007294.4(BRCA1): c.1953_1956del | NP_009225.1: p.Lys653SerfsTer47 | | BC, OVC, & gastric ca | | 10 | | 1 | | rs80357526 | | |  |  |
| **NM_007294.4(BRCA1): c.2641G>T** | **NP_009225.1: p.Glu881Ter** | | **BC, pancreatic ca, & gastric ca** | | **10** | | **1** | | **rs397508988** | | |  |  |
| NM_007294.4(BRCA1): c.4308_4309delTT | NP_009225.1: p.Ser1437CysfsTer3 | | BC, & thyroid ca | | 12 | | 1 | | no rs | | |  |  |
| NC_000017.11(BRCA1): g.(?_43082330)_(43082599_?)dup |  | | BC | | 12 | | 1 | | nsv3876777 | | |  |  |
| NC_000017.11(BRCA1): g.(?_43048992)_(43049260_?)del |  | | BC | | 21 | | 1 | | nsv5673039 | | |  |  |
| NC_000017.11(BRCA1): g.(?_43045584)_(43045853_?)del |  | | BC | | 23 | | 1 | | nsv4681747 | | |  |  |
| **Total** |  | |  | |  | | **12** | |  | | |  |  |
| ***BRCA2*** | | | | | | | | | | |  |  |  |
| **NM_000059.4(BRCA2): c.582G>A** | **NP_000050.3: p.Trp194Ter** | | **BC, & bladder ca** | | **7** | | **2** | | **rs80358810** | | |  |  |
| NM_000059.4(BRCA2): c.1261C>T | NP_000050.3: p.Gln421Ter | | BC, esophageal ca, & uterine ca | | 10 | | 2 | | rs80358419 | | |  |  |
| NM_000059.4(BRCA2): c.1705C>T | NP_000050.3: p.Gln569Ter | | BC | | 10 | | 1 | | no rs | | |  |  |
| NM_000059.4(BRCA2): c.3865_3868delAAAT | NP_000050.3: p.Lys1289AlafsTer3 | | BC, cervix ca, & throat ca | | 11 | | 1 | | rs80359412 | | |  |  |
| NM_000059.4(BRCA2): c.4554delA | NP_000050.3: p.Glu1518AspfsTer25 | | BC | | 11 | | 1 | | rs80359458 | | |  |  |
| **NM_000059.4(BRCA2): c.5771_5774delTTCA** | **NP_000050.3: p.Ile1924ArgfsTer38** | | **Male and female BC** | | **11** | | **4** | | **rs80359535** | | |  |  |
| NM_000059.4(BRCA2): c.6761_6762delTT | NP_000050.3: p.Phe2254TyrfsTer6 | | BC | | 11 | | 1 | | rs80359624 | | |  |  |
| NM_000059.4(BRCA2): c.7618-1G>A | NP_000050.3: p.? | | BC | | Intronic | | 1 | | rs397507389 | | |  |  |
| **NM_000059.4(BRCA2): c.7934delG** | **NP_000050.3: p.Arg2645AsnfsTer3** | | **BC, lung ca, uterine ca, & prostate cancer** | | **17** | | **4** | | **rs80359688** | | |  |  |
| NM_000059.4(BRCA2): c.8696_8712del | NP_000050.3: p.Gln2899LeufsTer2 | | BC | | 21 | | 1 | | no rs | | |  |  |
| NM_000059.4(BRCA2): c.8954-2A>G | NP_000050.3: p.? | |  | | Intronic | | 1 | | rs1135401928 | | |  |  |
| NM_000059.4(BRCA2): c.9154C>T | NP_000050.3: p.Arg3052Trp | | BC | | 24 | | 1 | | rs45580035 | | |  |  |
| NM_000059.4(BRCA2): c.9196C>T | NP_000050.3: p.Gln3066Ter | | BC, & prostate ca | | 24 | | 1 | | rs80359180 | | |  |  |
| NM_000059.4(BRCA2): c.9883C>T | NP_000050.3: p.Gln3295Ter | | BC | | 27 | | 1 | | rs80359247 | | |  |  |
| **Total** |  | |  | |  | | **22** | |  | | |  |  |

| ***Other genes*** | | | | | |  |
| --- | --- | --- | --- | --- | --- | --- |
| NM_000051.4(ATM): c.2638+2T>G | NP_000042.3: p.? | BC, pancreatic ca, & gastric ca | Intronic | 1 | no rs | |
| NM_000051.4(ATM): c.5212A>T | NP_000042.3: p.Lys1738Ter | BC, & uterine ca | 35 | 1 | no rs | |
| NM_000051.4(ATM): c.7517_7520del | NP_000042.3: p.Arg2506ThrfsTer3 | BC | 51 | 1 | rs587781905 | |
| NC_000011.10(ATM): g.(?_108364964)_(108365558_?)del | NP_000042.3: p.? | BC | 62 & 63 | 1 | nsv6112749 | |
| NM_000465.4(BARD1): c.390_391del | NP_000465.2: p.Ser131PhefsTer3 | BC | 4 | 1 | no rs | |
| NM_000465.4(BARD1): c.1216C>T | NP_000465.2: p.Arg406Ter | BC | 4 | 1 | rs377153250 | |
| NM_032043.3(BRIP1): c.751C>T | NP_114432.2: p.Arg251Cys | BC, leukemia, & bone ca | 7 | 2 | rs752309409 | |
| NM_007194.4(CHEK2): c.283C>T | NP_009125.1: p.Arg95Ter | BC, cervical ca, liver ca, pancreatic ca, bone ca, & colon ca | 2 | 2 | rs587781269 | |
| NM_007194.4(CHEK2): c.538C>T* | NP_009125.1: p.Arg180Cys | BC | 4 | 1 | rs77130927 | |
| NM_007194.4(CHEK2): c.1100delC | NP_009125.1: p.Thr367MetfsTer15 | BC | 12 | 1 | rs555607708 | |
| NM_024675.4(PALB2): c.3507_3508del | NP_078951.2: p.His1170PhefsTer19 | BC, & lung ca | 13 | 1 | rs587776428 | |
| NM_000546.6(TP53): c.158_163delGGTTCAinsAT | NP_000537.3: p.Trp53SerfsTer69 | BC | 4 | 1 | no rs | |
| **Total** |  |  |  | **14** |  | |

Abbreviations: BC, breast cancer; OVC, ovarian cancer; ca, cancer, * – variant confirmed to have a low but definite effect.
