# Supplementary material for: Implementation of multigene panel testing for breast and ovarian cancer in South Africa: A step towards excellence in oncology for the public sector
Source: Front Oncol. 2022 Dec 7;12:938561. doi: 10.3389/fonc.2022.938561 (PMC9768488; doi:10.3389/fonc.2022.938561)
Supplement: Supplementary file 4 [file Table_3.docx]

**TABLE S3:** Variants of unknown clinical significance identified for the South African cohort using the Oncomine BRCA Expanded panel.

| Variant | Protein | Cancer type in  index or family | Exon | *n* of patients with variant | rs number |
| --- | --- | --- | --- | --- | --- |
| NM_000051.4(ATM): c.149A>C | NP_000042.3: p.Lys50Thr | BC | 3 | 1 | no rs |
| NM_000051.4(ATM): c.1358C>T | NP_000042.3: p.Pro453Leu | BC | 10 | 2 | rs786204124 |
| NM_000051.4(ATM): c.1438T>A | NP_000042.3: p.Leu480Ile | BC | 10 | 3 | no rs |
| NM_000051.4(ATM): c.1924_1926delGAA | NP_000042.3: p.Glu642del | BC | 13 | 2 | rs876659575 |
| NM_000051.4(ATM): c.2887A>G | NP_000042.3: p.Met963Val | BC | 19 | 2 | rs374353016 |
| NM_000051.4(ATM): c.3078G>C | NP_000042.3: p.Trp1026Cys | BC | 21 | 1 | no rs |
| NM_000051.4(ATM): c.3857G>A | NP_000042.3: p.Cys1286Tyr | BC, & OVC | 26 | 1 | no rs |
| NM_000051.4(ATM): c.4279G>A | NP_000042.3: p.Ala1427Thr | BC, OVC, gastric ca, womb ca, prostate ca, & melanoma | 29 | 15 | rs2229021 |
| NM_000051.4(ATM): c.4362A>C | NP_000042.3: p.Lys1454Asn | BC | 29 | 1 | rs148993589 |
| NM_000051.4(ATM): c.6067G>A | NP_000042.3: p.Gly2023Arg | BC | 41 | 1 | rs11212587 |
| NM_000051.4(ATM): c.6176C>T | NP_000042.3: p.Thr2059Ile | BC | 42 | 2 | rs144761622 |
| NM_000051.4(ATM): c.6537T>G | NP_000042.3: p.Ile2179Met | BC | 45 | 1 | rs146243469 |
| NM_000051.4(ATM): c.6543G>T | NP_000042.3: p.Glu2181Asp | BC | 45 | 1 | rs138828590 |
| NM_000051.4(ATM): c.8558C>T | NP_000042.3: p.Thr2853Met | BC | 58 | 1 | rs141534716 |
| NM_000051.4(ATM): c.8672-6_8672-2del | NP_000042.3: p.? | BC, & endometrial ca | Intronic | 3 | no rs |
| NM_000465.4(BARD1): c.1153G>A | NP_000465.2: p.Asp385Asn | BC, prostate ca, & lung ca | 4 | 4 | rs587782436 |
| NM_000465.4(BARD1): c.1513G>A | NP_000465.2: p.Gly505Arg | BC, & womb ca | 6 | 3 | no rs |
| NM_000465.4(BARD1): c.2251C>T | NP_000465.2: p.Arg751Trp | BC | 11 | 1 | rs139785364 |
| NM_007294.4(BRCA1): c.503A>C | NP_009225.1: p.Lys168Thr | BC | 7 | 1 | rs273901743 |
| NM_007294.4(BRCA1): c.2267G>A | NP_009225.1: p.Arg756Lys | BC | 10 | 1 | rs975724885 |
| NM_007294.4(BRCA1): c.3328_3330delAAG | NP_009225.1: p.Lys1110del | BC | 10 | 1 | rs80358335 |
| NM_000059.4(BRCA2): c.2240A>G | NP_000050.3: p.Glu747Gly | BC | 11 | 1 | rs397507283 |
| NM_000059.4(BRCA2): c.3950C>G | NP_000050.3: p.Thr1317Ser | BC | 11 | 1 | no rs |
| NM_000059.4(BRCA2): c.9501+3A>T | NP_000050.3: p.? | BC, pancreas ca, colon ca, & leukemia | Intronic | 4 | rs61757642 |
| NM_000059.4(BRCA2): c.9875C>T | NP_000050.3: p.Pro3292Leu | BC, & Prostate ca | 27 | 10 | rs56121818 |
| NM_000059.4(BRCA2): c.9976A>T | NP_000050.3: p.Lys3326Ter | BC, pancreas ca, colon ca, & leukemia | 27 | 6 | rs11571833 |
| NM_032043.3(BRIP1): c.1795-12_1795-10del | NP_114432.2: p.? | OVC | Intronic | 1 | rs1475317357 |
| NM_032043.3(BRIP1): c.2477A>G | NP_114432.2: p.Asn826Ser | BC | 17 | 1 | rs760127237 |
| NM_016507.4(CDK12): c.3349G>A | NP_057591.2: p.Ala1117Thr | OVC | 13 | 1 | rs1414877781 |
| NC_000017.11(CDK12): g.(?_39462032) (39494723 ?)dup | NP_057591.2: p.? | BC | 1-5 | 1 | no nsv (dbVar nr) |
| NM_007194.4(CHEK2): *77A>TA | NP_009125.1: p.? | BC | 1 (5’ UTR) | 4 | no rs |
| NM_007194.4(CHEK2): c.164C>T | NP_009125.1: p.Ser55Phe | BC | 2 | 1 | rs765799649 |
| NM_007194.4(CHEK2): c.538C>T | NP_009125.1: p.Arg180Cys | BC, cervix ca, liver ca, pancreatic ca, & bone ca | 4 | 7 | rs77130927 |
| NM_007194.4(CHEK2): c.556A>C | NP_009125.1: p.Asn186His | BC, prostate ca, & womb ca | 4 | 4 | rs146198085 |
| NM_007194.4(CHEK2): c.1217G>A | NP_009125.1: p.Arg406His | BC | 12 | 1 | rs200649225 |
| NM_033084.6(FANCD2): c.*42A>G | NP_149075.2: p.? | BC | 43 | 1 | rs375632206 |
| NM_033084.6(FANCD2): c.3963+8C>T | NP_149075.2: p.? | BC | Intronic | 1 | rs201623111 |
| NM_033084.6(FANCD2): c.4067A>G | NP_149075.2: p.His1356Arg | BC | 42 | 1 | rs1348547984 |
| NM_005591.4(MRE11): c.256G>A | NP_005582.1: p.Asp86Asn | BC | 4 | 1 | rs763902512 |
| NM_005591.4(MRE11): c.1559G>A | NP_005582.1: p.Arg520His | BC | 14 | 1 | rs753148077 |
| NM_005591.4(MRE11): c.1948G>C | NP_005582.1: p.Asp650His | BC, cervical ca, & prostate ca | 18 | 9 | no rs |
| NM_002485.5(NBN): c.1398-10T>A | NP_002476.2: p.? | BC | Intronic | 1 | no rs |
| NM_002485.5(NBN): c.1405G>T | NP_002476.2: p.Asp469Tyr | BC, throat ca, & prostate ca | 11 | 3 | rs148205441 |
| NM_024675.4(PALB2): c.23C>T | NP_078951.2: p.Pro8Leu | BC, & uterine ca | 1 | 4 | rs150390726 |
| NM_024675.4(PALB2): c.474G>C | NP_078951.2: p.Gln158His | BC, & womb ca | 4 | 5 | rs878855119 |
| NM_002717.4(PPP2R2A): c.18A>G | NP_002708.1: p.Gly16= | BC | 2 | 1 | rs1413011339 |
| NM_133509.5(RAD51B): c.*59_*60dup | NP_598193.2: p.? | OVC | 13 (3’ UTR) |  | rs1275625911 |
| NM_133509.5(RAD51B): c.436G>A | NP_598193.2: p.Ala146Thr | BC, stomach ca, & pancreas ca | 5 | 3 | rs200741476 |
| NM_133509.5(RAD51B): c.914A>T | NP_598193.2: p.Asn305Ile | BC, uterine ca, gastric ca, prostate ca, & throat ca | 9 | 10 | no rs |
| NM_133509.5(RAD51B): c.1094C>G | NP_598193.2: p.Pro365Arg | OVC | 11 | 1 | rs28908468 |
| NM_001142548.2(RAD54L): c.1393_1395delGAT | NP_001136020.1: p.Asp465del | BC, & colon ca | 14 | 2 | rs773026168 |
| NM_001142548.2(RAD54L): c.1523C>T | NP_001136020.1: p.Thr508Ile | BC | 15 | 2 | rs573607021 |
| NM_001142548.2(RAD54L): c.2026C>T | NP_001136020.1: p.His76Tyr | BC | 18 | 4 | rs751010909 |
| NM_000546.6(TP53): c.116C>T | NP_000537.3: p.Ala39Val | BC | 4 | 1 | rs1353016807 |

Abbreviations: BC, breast cancer; OVC, ovarian cancer; ca, cancer, * – variant located in the untranslated region (UTR)
