# Supplementary material for: Implementation of multigene panel testing for breast and ovarian cancer in South Africa: A step towards excellence in oncology for the public sector
Source: Front Oncol. 2022 Dec 7;12:938561. doi: 10.3389/fonc.2022.938561 (PMC9768488; doi:10.3389/fonc.2022.938561)
Supplement: Supplementary file 5 [file Table_4.docx]

**TABLE 4:** Genes included in the Oncomine BRCA Expanded NGS panel and associated absolute risks for developing breast and ovarian cancer based on relevant literature and the NCCN 2022v2 guidelines.

| Gene | Breast cancer lifetime/absolute risk*  (%) | Ovarian cancer lifetime/absolute risk* (%) | Odd ratios* | Proposed management options depending on variant penetrance in relation to the molecular pathway | Role of the protein and associated therapeutic options (guidelines and literature-based) | References |
| --- | --- | --- | --- | --- | --- | --- |
| *ATM* | 25–30% | <3% | BC: 2.0–2.3  OVC: 2.85 | - *BRCA1/2-*equivalent breast surveillance by screening alone and magnetic resonance imaging from age 40 years - Insufficient evidence for risk-reduction surgery - Managed based on family history | - Involved in cell cycle control, apoptosis, gene regulation, oxidative stress, and telomere maintenance - Are mostly endocrine-positive, dedifferentiated and more aggressive - Treatment with PARP inhibitors such as Olaparib - Treatment with CDK4/6 inhibitors in combination with endocrine treatment such as Palbociclib, Ribociclib and Abemaciclib, depending on breast cancer type | - Easton et al. (2015) - Lima et al. (2019) - Lu et al. (2019) - Li et al. (2020) - Dorling et al. (2021) - NCCN 2022v2 |
| *BARD1* | 15–40% | No established association | BC: 2.0–2.5 | - *BRCA1/2-*equivalent breast surveillance - Insufficient evidence for risk-reduction surgery - Managed based on family history | - Treatment with PARP inhibitors such as Lynparza (Olaparib) for advanced ovarian cancer in combination with bevacizumab as maintenance therapy after first-line platinum chemotherapy - Zejula, Rubraca and Lynparza for maintenance therapy after treatment of recurrent epithelial ovarian cancer, fallopian tube or primary peritoneal cancer who are in complete or partial response to platinum-based chemotherapy | - Lima et al. (2019) - Dorling et al. (2021) - NCCN 2022v2 |
| *BRCA1* | >60% | 44% | BC: 9.3–22  OVC: 3.27 | - *BRCA1/2* surveillance commencing 40 years, earlier depending on family history - Risk-reducing mastectomy - Bilateral oophorectomy after child-bearing | - Regulator of DNA repair, transcription, and cell death in reply to DNA damage - Play a crucial role in maintaining DNA integrity - Effective platinum-based chemotherapy in combination with PARP inhibitors-based therapy such as Olaparib - PARP inhibitors-based therapy alone | - Lima et al. (2019) - Dorling et al. (2021) - NCCN 2022v2 |
| *BRCA2* | >60% | 13–29% | BC: 5.3–8.1 | - *BRCA1/2* surveillance commencing 40 years, earlier depending on family history - Risk-reducing mastectomy - Risk-reducing oophorectomy 10 later than BRCA1 carriers | - Regulator of DNA repair, transcription, and cell death in reply to DNA damage - Play a crucial role in maintaining DNA integrity - Effective platinum-based chemotherapy in combination with PARP inhibitors-based therapy such as Olaparib - PARP inhibitors-based therapy alone | - Lima et al. (2019) - Dorling et al. (2021) - NCCN 2022v2 |
| *BRIP1* | Insufficient data to define | >10% | OVC: 4.94 | - *BRCA1/2-*equivalent breast surveillance - For breast cancer, managed based on family history - For ovarian cancer, consider risk-reducing oophorectomy starting at age 45 – 50 years | - *BRCA1/2-*equivalent breast surveillance - Part of the Fanconi gene family - Is a DNA-dependent helicase that interacts with BRCA1 to preserve genetic stability - PARP inhibitors-based therapy | - Suszynska, Rataiska & Kozlowski (2020) - NCCN 2022v2 |
| *CHEK2* | 25–30% | No established association | BC: 1.5–2.6 | - *BRCA1/2-*equivalent breast surveillance - Insufficient evidence for risk-reduction surgery - Managed based on family history | - Plays a crucial role in regulation of p53 function and BRCA1 - Stops the cell from dividing - Neoadjuvant chemotherapy - Effectiveness of systemic therapy for breast cancer patients is limited | - Easton et al. (2015) - Liu et al. (2015) - Apostolou & Papasotiriou (2017) - Lima et al. (2019) - Lu et al. (2019) - Dorling et al. (2021) - NCCN 2022v2 |
| *PALB2* | 41–60%, modified by familial factors | 3–5% | BC: 5.0–9.0  depending on age  OVC: 3.34 | - *BRCA1/2-*equivalent breast surveillance - Annual mammogram commencing at age 30 - Risk-reducing mastectomy is recommended guided by personalized risk estimates - No ovarian cancer surveillance - Risk-reducing salpingo-oophorectomy should only be considered before the age of 50 | - Localizes BRCA2 during homologous recombination and double-sstrand breast repair - Together, the proteins assist with the regulation of cell growth and division of cells - *BRCA1/2-*equivalent breast surveillance - Treatment with PARP inhibitors such as Olaparib and Talazoparib for breast cancer | - Antoniou et al. (2014) - Keung et al. (2019) - Lu et al. (2019) - Yang et al. (2020) - Dorling et al. (2021) - Tischkowitz et al. (2021) - NCCN 2022v2 |
| *TP53* | >60%, especially for triple-positive disease | 18.5% | BC: 3.06 | - Mastectomy is recommended over lumpectomy to avoid adjuvant breast radiotherapy - Risk-reducing surgery advocated due to the high contralateral breast cancer risk | - Protein is responsible for various stress signals and suppression of cellular transformation via mediating cell-cycle arrest, the cellular response against oncogenic stress, and cell repair and apoptosis - No actionable drugs - Less responsive to low-dose radiation - High risk of new malignancies induced by radiotherapy | - Evans et al. (2006) - Schon & Tischkowitz (2018) - Lima et al. (2019) - Lu et al. (2019) - Montalban-Bravo et al. (2020) - Dorling et al. (2021) - NCCN 2022v2 |

*Risk estimates and odds ratios are dependent on the variant type and functional domains

NCCN Genetic/familial high-risk assessment: breast, ovarian and pancreatic cancer 2002v2 – <https://www.nccn.org/professionals/physician_gls/pdf/genetics_bop.pdf>

NCCN Clinical Practise Guidelines in Oncology: Breast cancer Version 4.2022 – <https://www.nccn.org/professionals/physician_gls/pdf/breast.pdf>

NCCN Clinical Practise Guidelines in Oncology: Ovarian cancer including fallopian tube cancer and primary peritoneal cancer Version 4.2022 – <https://www.nccn.org/professionals/physician_gls/pdf/ovarian.pdf>
